# Differential Expression and Release of Activin A and Follistatin in Chronic Rhinosinusitis with and without Nasal Polyps

**DOI:** 10.1371/journal.pone.0128564

**Published:** 2015-06-01

**Authors:** Yucheng Yang, Nan Zhang, Koen Van Crombruggen, Feng Lan, Guohua Hu, Suling Hong, Claus Bachert

**Affiliations:** 1 Department of Otorhinolaryngology, the First Affiliated Hospital, Chongqing Medical University, Chongqing, China; 2 Upper Airway Research Laboratory, Department of Otorhinolaryngology, Ghent University, Ghent, Belgium; 3 Division of Nose, Throat and Ear Diseases, Clintec, Karolinska Institute, Stockholm, Sweden; Okayama University, JAPAN

## Abstract

**Background:**

Chronic rhinosinusitis with (CRSwNP) and without nasal polyps (CRSsNP) should be regarded as distinct clinical entities based on differential inflammatory mediator and remodeling profiles. Activin A, a member of the TGF-β superfamily, plays an important role in inflammation and remodeling in the lower airways, although its expression and release in the upper airways remain undescribed.

**Objective:**

To investigate the expression of activin A and its inhibitor follistatin in nasal tissue samples from CRSsNP and CRSwNP patients, and to monitor the spontaneous release of these molecules in a human mucosal model.

**Methods:**

Protein levels were determined using ELISA for activin A, follistatin, TGF-β1 and indicator proteins (IL-5, ECP, IFNγ) in 13 CRSsNP, 23 CRSwNP, and 10 control samples. The spontaneous release rate and the release ratios of activin A, follistatin and TGF-β1 were determined in 9 CRSsNP and 7 CRSwNP tissue fragments cultured ex-vivo. The induction of activin A and TGF-β1 by one another was studied in 7 CRSsNP tissue fragments cultured ex-vivo.

**Results:**

Significantly higher concentrations of activin A, follistatin, TGF-β1, and IFNγ were observed in CRSsNP compared with CRSwNP samples, whereas the concentrations of IL-5 and ECP were significantly lower. Follistatin was positively and linearly correlated with activin A in CRSsNP and CRSwNP. Activin A, follistatin and TGF-β1 were all spontaneously released by the samples, although the relative ratios released by tissue fragments from CRSsNP and CRSwNP samples were significantly different, with a higher follistatin/activin A-ratio and a follistatin/TGFß1-ratio (with less overall TGF-β1) in CRSwNP than in CRSsNP. Furthermore, TGF-β1 enhanced activin A secretion in CRSsNP tissue fragments cultured ex-vivo.

**Conclusion:**

The differences in tissue concentrations and spontaneous release rates for activin A and follistatin in different CRS samples support the hypothesis that CRSsNP and CRSwNP are two distinct disease entities with respect to remodeling patterns.

## Introduction

Chronic rhinosinusitis (CRS) is a common health problem with an overall prevalence of 10.9% in Europe by EP(3)OS criteria [1], and it is currently classified into two major subgroups: chronic rhinosinusitis with nasal polyps (CRSwNP) and chronic rhinosinusitis without nasal polyps (CRSsNP) [2]. Evidence suggests that CRSwNP and CRSsNP should be regarded as distinct clinical entities, based on different inflammatory mediator and remodeling profiles [3–5]. CRSwNP is characterized by dysregulation of regulatory T (Treg) cells and inflammatory cells, coincident with intense edematous stroma, albumin deposition and pseudocyst formation; by contrast, CRSsNP is characterized by normal Treg cell number and migration capacity and significantly less severe inflammatory mucosal reactions, coincident with fibrosis, basement membrane thickening and goblet cell hyperplasia [6, 7]. However, the exact mechanism of inflammation and remodeling in CRS has not been elucidated.

Recently, it has been found that the transforming growth factor beta (TGF-β) superfamily plays an important pathophysiological function in airway diseases, including CRS [7]. The TGF-β superfamily consists of more than 33 members, all of which have a similar prodomain fold [8], and they control numerous physiological processes, including wound healing, tissue regeneration and immunity [9]. TGF-β1, an important member of this superfamily, and its signaling transduction pathways are up-regulated in CRSsNP and down-regulated in CRSwNP [10]. Recent evidence suggests that TGF-β1 is involved in the very early events of respiratory disease as well as in late persistent disease, both in inflammatory and remodeling processes [11]. Activin A, another important member of the TGF-ß superfamily, utilizes similar signaling transduction pathways as TGF-β1, including Smad-dependent and Smad-independent pathways [9]. Generally, TGF-β1 is an activator of tissue fibrosis and a potent inhibitor of inflammation. Thus, it is possible that activin A also functions to activate tissue repair programs while modulating the inflammatory response.

A growing body of evidence suggests that the deregulation of activin signaling contributes to pathologic conditions such as chronic inflammation, fibrosis and carcinogenesis [12–15]. Follistatin, also known as activin-binding protein, is an endogenously produced protein that binds activin A with high affinity and inhibits its bioactivity. The primary function of follistatin is the binding and bioneutralization of members of the TGF-β superfamily, and activin A in particular [12]. Moreover, in lower airway disease, both activin A and TGF-β1 have been implicated in airway inflammation and remodeling, and their signaling pathways are activated upon allergen provocation in asthma [14]. Changes in the balance of activin A and follistatin – which may be a determinant of the severity of allergic inflammation or tissue phenotypic shift – have also been observed in asthma [15]. We found that TGF-β1 serves as an important switch for different inflammatory and remodeling patterns in sinus disease [7, 10]. In addition to detecting several important inflammatory and remodeling cytokines, such as IFNγ, IL-5, and eosinophil cationic protein (ECP), the aim of the present study was to analyze the expression and spontaneous release of activin A and follistatin, as well as the interaction between activin A and TGF-β1 in CRS. The study was also designed to test whether activin A expression is increased in CRSsNP and is associated with increased TGF-β1 levels, as well as whether the production of follistatin may be involved in the anti-fibrotic milieu in CRSwNP, consistent with the hypothesis that CRSsNP and CRSwNP are two distinct disease entities.

## Materials and Methods

### Patients and sample collection

The study subjects were selected at the Department of Otorhinolaryngology of Ghent University Hospital, Belgium. The diagnosis of sinus disease was carried out according to European Position Paper on Rhinosinusitis and Nasal Polyps guidelines (EPOS) [2], which are based on history, clinical examination, nasal endoscopy, and sinus CT scans. General exclusion criteria were based on the EPOS definition for research. None of the subjects received anti-histamines, anti-leukotrienes, oral or intranasal decongestants, or intranasal anti-cholinergics during the 2 weeks prior to surgery. A washout period was maintained for 4 weeks before surgery for oral and topical corticosteroids and antibiotics. Females who were pregnant or breast-feeding were excluded from the study. The ethics committee of the Ghent University Hospital approved the study, and all patients gave their written informed consent prior to inclusion in the study.Samples from patients with CRSsNP (n = 13; median age, 39 years; range, 20–81 years; 6 women, 7 men) and CRSwNP (n = 23; median age, 49 years; range, 19–71 years; 10 women, 13 men) were obtained during functional endoscopic sinus surgical procedures. Inferior turbinate samples from patients without sinus disease undergoing septoplasty or rhinoseptoplasty were collected as controls (n = 10; median age, 29 years; range, 18–60 years; 4 women, 6 men). For CRSsNP, the tissue samples were collected from ethmoidal mucosa. For CRSwNP, samples of ethmoidal polyp tissue were used. All patients underwent a skin prick test (SPTs) against the European standard panel of 14 inhalant allergens. Negative and positive controls (10 mg/mL histamine solution) were included with each SPT. None of the control subjects had a history of asthma or a positive SPT. One of the patients with CRSsNP and 7 of the patients with CRSwNP had a positive SPT. In the CRSwNP group, 5 of the 23 patients had a history of asthma, and 2 patients reported aspirin intolerance (Table 1).

**Table 1:** Patient’s details.

	Control	CRSsNP	CRSwNP	P values
No. of patients	10	13	23	
Age (y) median (range)	29(18–60)	39(20–81)	49(19–71)	#
No. of female/male	4/6	6/7	10/13	0.957
Skin prick test–positive (n/N)	0/10	1/13	7/23	0.070*
Asthma in history (n/N)	0/10	0/13	5/23	0.087*
Aspirin intolerance (n/N)	0/10	0/13	2/23	0.711*

# p = 0.0097 (CRSwNP vs Control) was obtained by ANOVA. Constituent ratio was compared by crosstabs. Gender comparison was done by Chi-square test

*Fisher exact test. P <0. 05 was considered statistically significant.

The nasal tissues collected during surgery were immediately transported to the laboratory, snap frozen in liquid nitrogen, and stored at -80°C until analysis by ELISA. Remaining tissues from specific subjects were used for ex vivo tissue stimulation experiments.

### Measurements of mediators in tissue homogenates

The frozen tissues were thawed, weighed, homogenized and centrifuged as described previously [16]. The samples were then assayed for activin A, follistatin, total TGF-β1, and other cytokines (IFNγ, IL-5, ECP) using commercially available ELISA kits from R&D Systems (Minneapolis, Minnesota, USA) with the appropriate dilution factor and according to kit instructions. All data were expressed as picogram per milliliter. For TGF-β1, acid was added during the ELISA procedure, resulting in physicochemical activation of latent TGF-β1. Total TGF-β1 concentrations were reported, including both active and latent forms.

### Immunofluorescence assay

The localization of Activin A and follistatin in nasal tissue was assessed using immunofluorescence microscopy. Cryo-sections (5μm) of nasal tissue were firstly incubated with 0.2% Triton X-100 at room temperature (RT) for 10 min, were then blocked with 0.5% bovine serum albumin for 30 min. The samples were incubated with anti-ActivinA (7.5μg/ml) or anti-follistatin (7.5μg/ml) primary polyclonal goat antibodies (R&D) for 2 h at RT, washed, and incubated with the corresponding rabbit anti-goat IgG (R&D) (1:200) for 1 h at 37°C. The nuclei were labeled with 4,6-diamidino-2-phenylindole (DAPI), and the sections were visualized by fluorescence microscopy.

### Preparation and stimulation of human nasal tissue ex vivo

Among the patients included, 9 CRSsNP, 7 CRSwNP patients and 7 controls without positive SPT, asthma or aspirin intolerance, as well as less than 65 years old, were selected for ex-vivo culture experiments. The nasal tissue explants were prepared as described previously [16, 17]. Briefly, the human nasal mucosa and submucosa were thoroughly fragmented in RPMI-1640 culture medium (Sigma-Aldrich, Bornem, Belgium) containing 2 mM l-glutamine (Invitrogen, Merelbeke, Belgium), 50 IU/ml penicillin, 50 mg/ml streptomycin (Invitrogen) and 0.1% BSA (Sigma-Aldrich). Tissue fragments were then weighed, resuspended at 0.04 g tissue/ml in culture medium and pre-incubated for 1 h at 37°C and 5% CO2. The tissue fragments were resuspended in the appropriate amount of culture medium, and 1 ml of the fragment suspension was dispensed per well in a 12-well plate (BD Falcon, VWR, Leuven, Belgium). Due to increased expression levels in CRSsNP, the CRSsNP fragment suspensions were selected and stimulated with either culture medium (negative control) for 1 h, 4 h and 24 h or with TGF-β1 or Activin A (Sigma-Aldrich N.V./S.A, Bornem, Belgium) for 24 h. Supernatants were separated by centrifugation and stored immediately at -20°C until analysis by ELISA.

### Quantification of mediators in the supernatants of stimulated tissue fragments

The concentrations of activin A, follistatin, and TGF-β1 were measured in tissue supernatants obtained after stimulation using ELISA kits from R&D Systems (Minneapolis, Minnesota, USA), according to the manufacturer's instructions.

### Statistical analysis

Statistical analysis was performed using the MEDCALC version 13.1.2.0 software program (Schoonjans F, Mariakerke, Belgium). Normal distributed data are expressed as mean values (±SD), and for variables with a non-Gaussian distribution, values are expressed as median and inter-quartile ranges (25th–75th percentile). Statistical analysis was performed by using the Wilcoxon test (for paired comparisons). The Mann-Whitney U test was used for between-group (unpaired) comparisons. Constituent ratio was compared by crosstabs. Linear correlation analysis was performed between two variables of activin A, follistatin and TGF-β1 in each group. Exact p values were reported. The significance level was set at α = 0.05.

## Results

### Protein expression in nasal tissue

**Fig 1:**
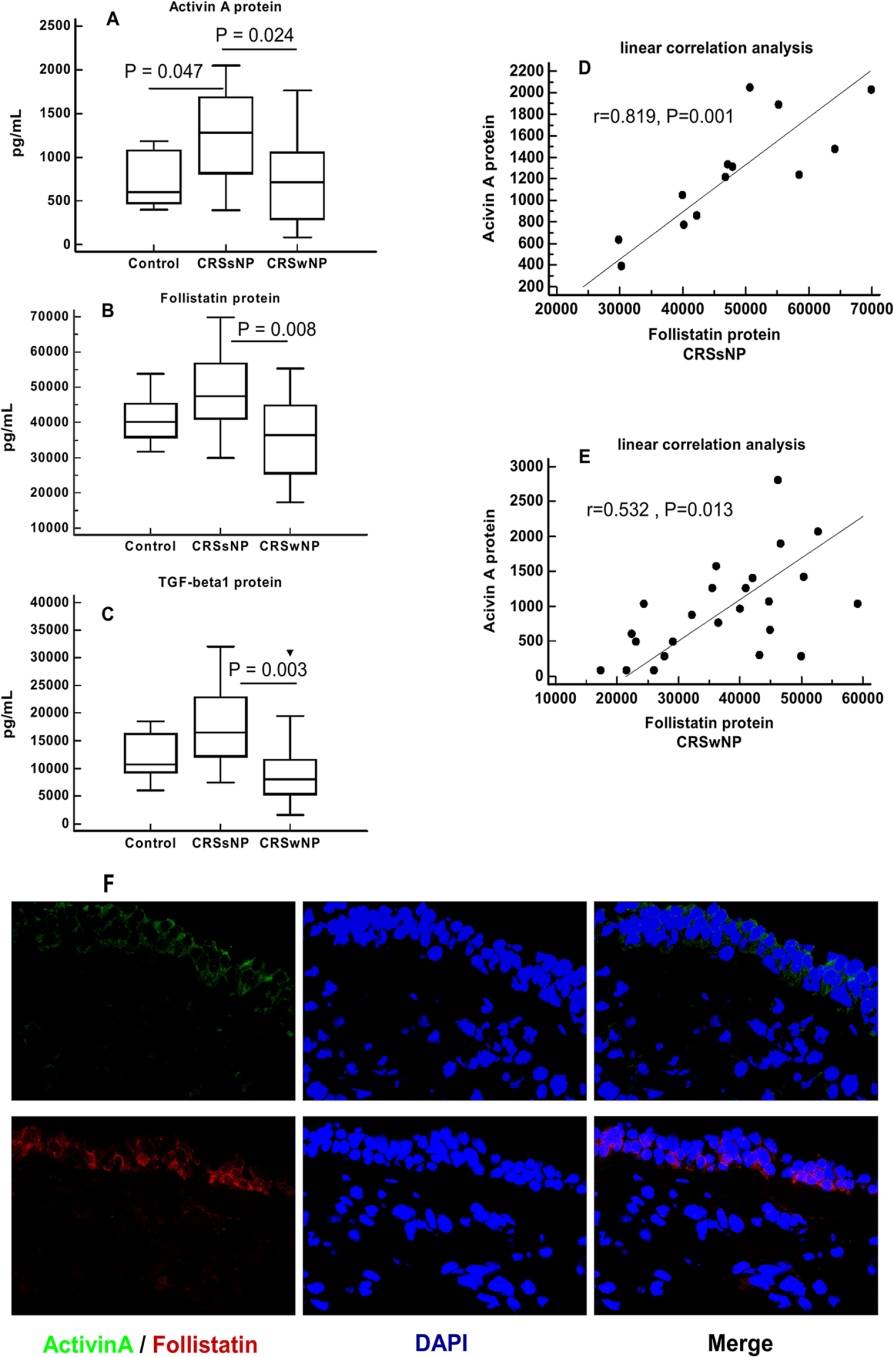
Protein expression and linear correlation (D, E) of activin A (A), Follistatin (B) and TGF-beta1 (C) in nasal tissue from CRSsNP (n = 13), CRSwNP (n = 23) and Control patients (inferior turbinate) (n = 10). The figures show that the expression of all three proteins (Activin A, Follistatin and TGF-ß1) were higher in CRSsNP than in CRSwNP; Follistatin protein was positively correlated with activin A protein in CRSsNP and in CRSwNP. Significance is indicated by P value. Immunofluorescence staining (F) showed both Activin A (green fluorescence) and follistatin (red fluorescence) were mainly expressed in the cytoplasm of nasal epithelial cells. ×630.

**Fig 2:**
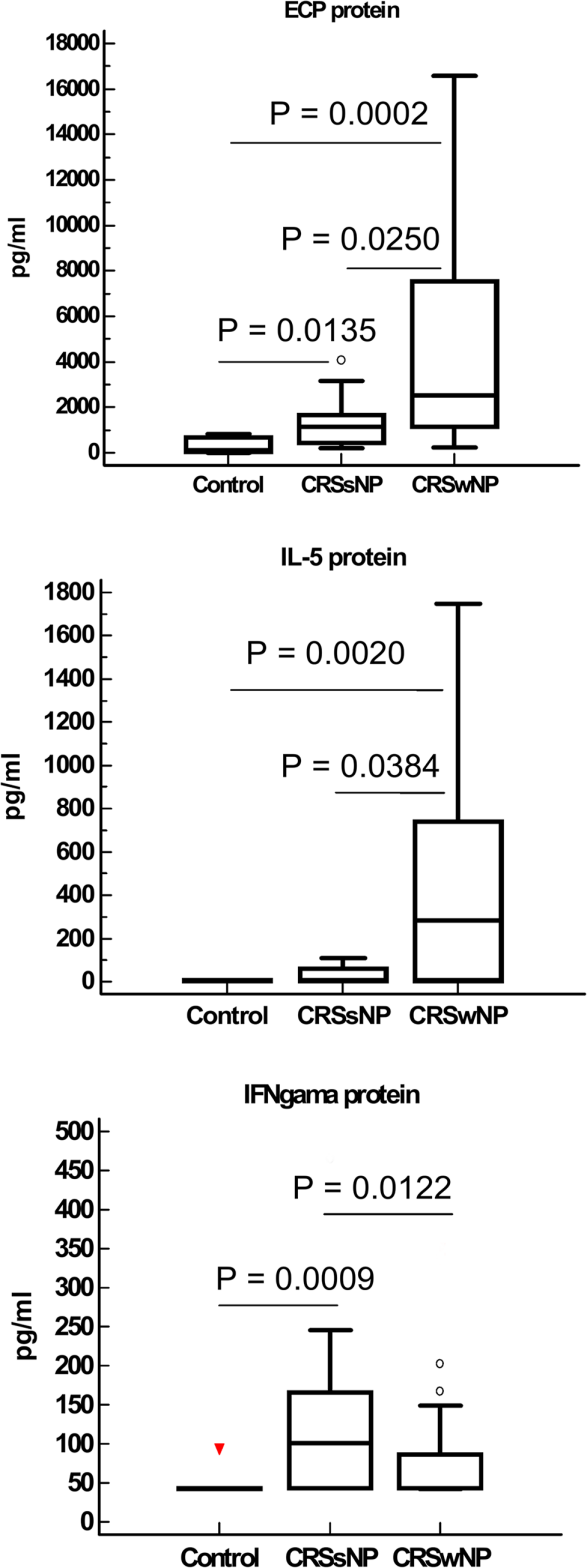
Protein expression of ECP, IL-5 and IFNγ in nasal tissue from CRSsNP (n = 13), CRSwNP (n = 23) and Control patients (inferior turbinate) (n = 10). IFNγ protein expression was significantly higher and IL-5 and ECP expression was significantly lower in CRSsNP compared with CRSwNP. Significance is indicated by P value.

Tissue homogenates were tested by ELISA. Activin A protein expression was significantly higher in CRSsNP compared with CRSwNP (p = 0.024) and controls (p = 0.047). Significantly increased expression of follistatin protein in CRSsNP relative to CRSwNP (p = 0.008) was observed. Similarly, the expression of TGF-β1 protein was significantly higher in CRSsNP than in CRSwNP (p = 0.003). A linear correlation analysis was carried out between these parameters, which revealed that follistatin protein levels were positively correlated with activin A protein levels in CRSsNP (r = 0.819, p = 0.001) and in CRSwNP (r = 0.532, p = 0.013) (Fig 1). Immunofluorescence staining (Fig 1F) showed Activin A and follistatin were mainly expressed in the cytoplasm of nasal epithelial cells. IFNγ protein expression was significantly higher and IL-5 and ECP expression was significantly lower in CRSsNP compared with CRSwNP (Fig 2).

### Spontaneous release in nasal tissue fragments

**Fig 3:**
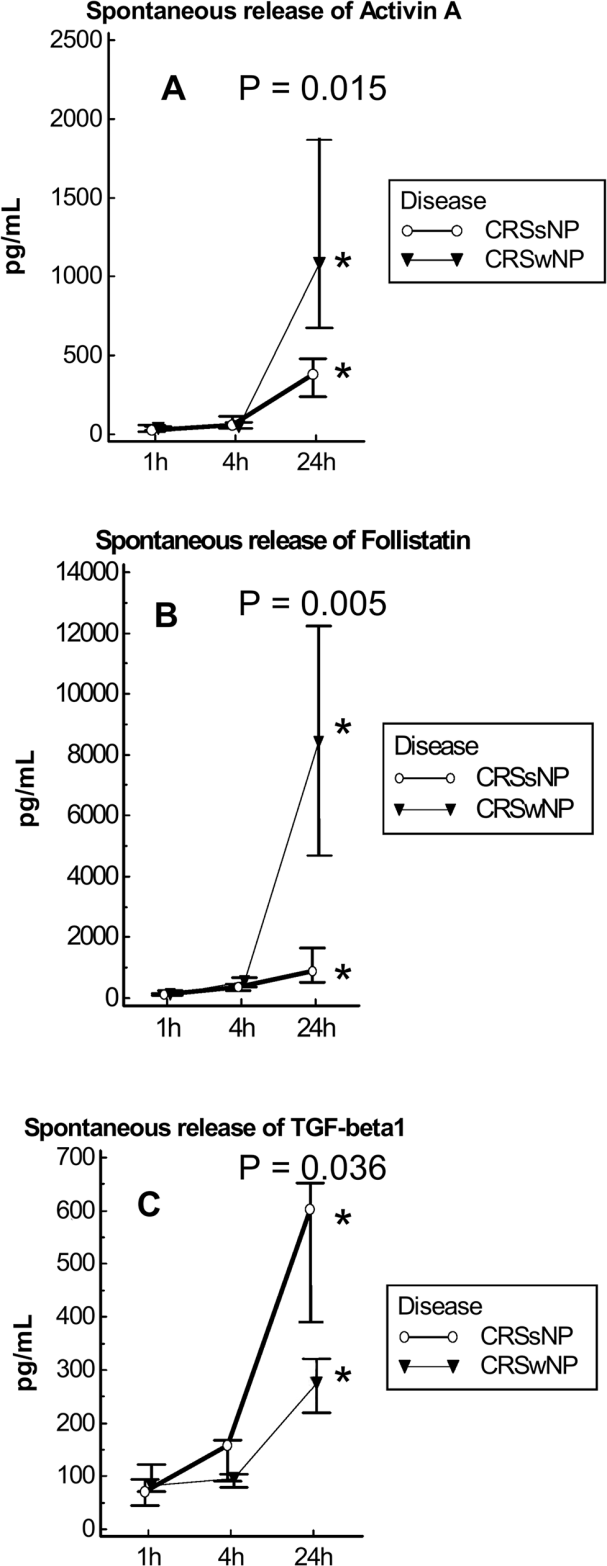
Spontaneous release of activin A (A), Follistatin (B) and TGF-beta1 (C) in supernatants of nasal tissue fragments from CRSsNP (n = 9) and CRSwNP patients (n = 7). The release of activin A (p = 0.015) (Fig 3A) and follistatin (p = 0.005) (Fig 3B) was higher in CRSwNP than in CRSsNP subjects; in contrast, the release of TGF-β1 (p = 0.036) (Fig 3C) was lower in CRSwNP than in CRSsNP at 24h. *P<0.05 compared to 1h and 4h.

Nasal tissue fragments from CRSsNP (n = 9) and CRSwNP (n = 7) were plated in RPMI without stimulation. The supernatants were evaluated by ELISA at 1, 4, and 24 h (Fig 3). Compared with the 1 h time point, the release of activin A, follistatin and TGF-β1 was significantly enhanced at 4 h (p<0.05) and 24 h (p<0.01) in CRSsNP and at 24 h (p<0.05) in CRSwNP. At 24 h, the release of activin A (p = 0.015) (Fig 3A) and follistatin (p = 0.005) (Fig 3B) was higher in CRSwNP than in CRSsNP; in contrast, the release of TGF-β1 (p = 0.036) (Fig 3C) was lower in CRSwNP than in CRSsNP.

### Differential ratios of follistatin/activinA and follistatin/TGF-β1 in nasal tissue and supernatants

**Fig 4:**
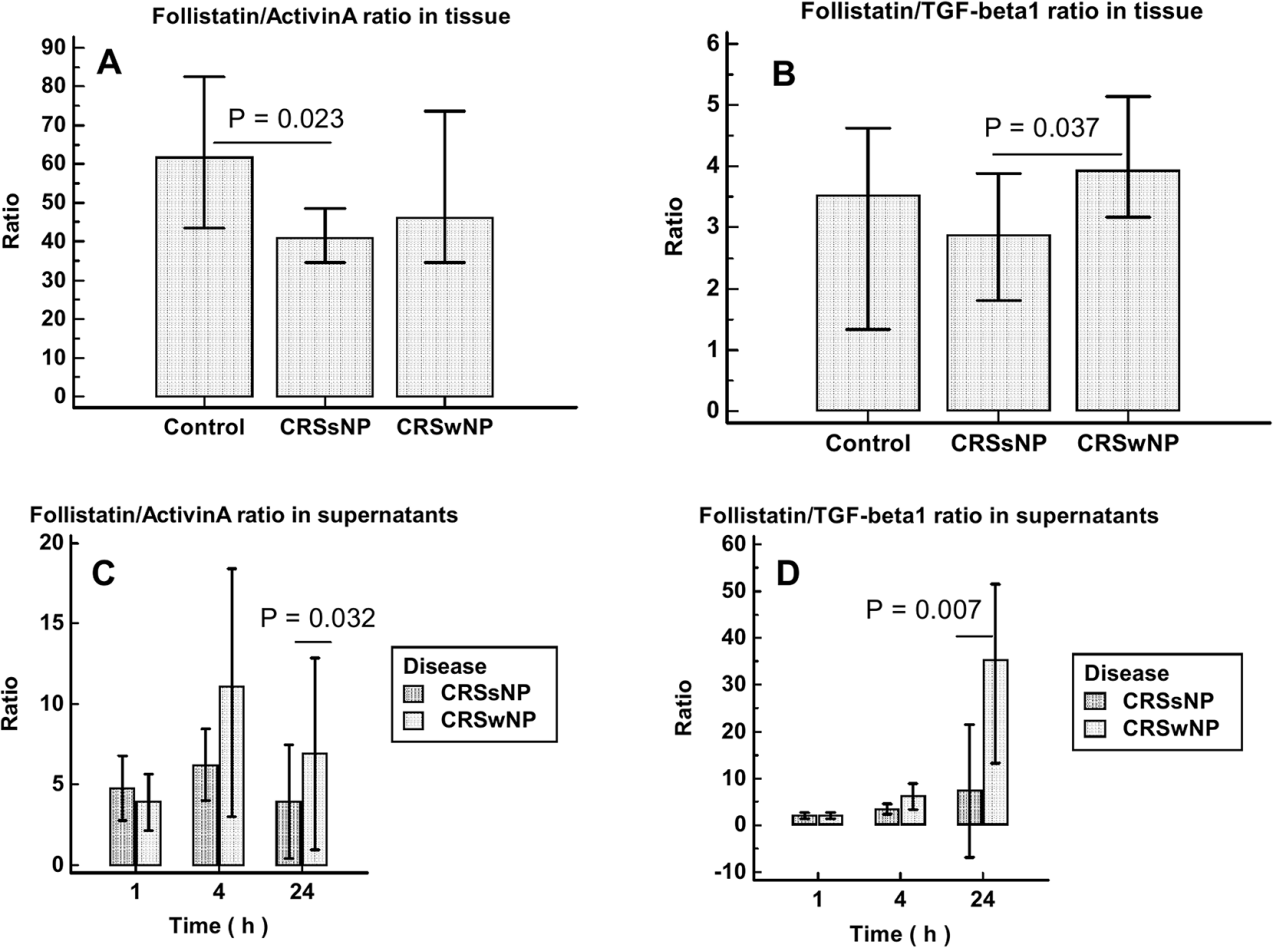
The ratios of Follistatin/ActivinA and Follistatin/TGF-beta1 in nasal tissue and supernatants. There was an imbalance between pro-fibrotic (TGF-beta1, activin A) and anti-fibrotic (follistatin) mediators in CRSsNP (n = 9) and CRSwNP (n = 7). Statistical analysis was performed by using the Wilcoxon test (for paired comparisons). Significance is indicated by P value.

To elucidate the relationship between follistatin, activin, and TGF-β1 in the nasal tissue samples and supernatants, the different ratios of follistatin/ActivinA and follistatin/TGF- β1 in nasal tissue homogenates and supernatants at 24 h were calculated and compared (Fig 4). In the tissue, the follistatin/activinA ratio was lower in CRSsNP than in the controls (p = 0.023) but was similar in CRSsNP and CRSwNP (Fig 4A). In addition, the follistatin/TGF- β1 ratio was lower in CRSsNP than in CRSwNP (p = 0.037) (Fig 4B). In the supernatants at 24 h, both the follistatin/activinA (p = 0.032) (Fig 4C) and follistatin/TGF- β1 (p = 0.007) (Fig 4D) ratios were lower in CRSsNP than in CRSwNP. An imbalance between pro-fibrotic (TGF- β1, activin A) and anti-fibrotic (follistatin) mediators was apparent in CRSsNP and CRSwNP.

### TGF-β1 enhances activin A secretion in CRSsNP tissue fragments ex vivo

**Fig 5:**
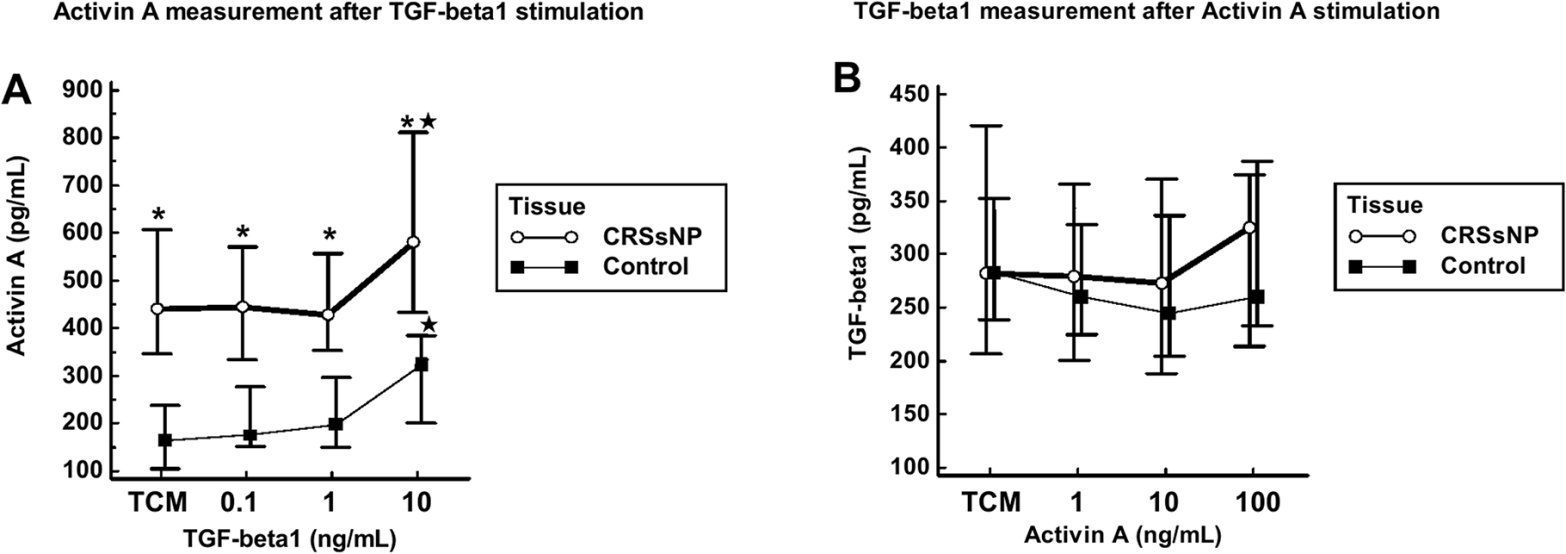
The induction of activin A and TGF-β1 by one another in nasal tissue fragments from CRSsNP (n = 7) and control patients (inferior turbinate) tissue (n = 7). The CRSsNP and control fragment suspensions were stimulated with TGF-β1 (0, 0.1, 1, 10 ng/mL) or Activin A (0, 1, 10, 100 ng/mL) for 24 h. Activin A and TGF-β1 were tested in supernatants by ELISA. The figures show that activin A was induced by TGF-beta1 in CRSsNP and controls, but TGF-ß1 was not induced by activin A. *p<0.05 compared to controls; ★ p<0.05 compared to 0 ng/ml.

To test the stimulatory effects of the autocrine factors TGF-β1 and activin A in CRSsNP tissue, we examined how they influenced the secretion of one another in nasal tissue fragments (Fig 5). Compared with controls, activin A secretion was higher in CRSsNP under each condition (p<0.05). Compared with 0 ng/ml, activin A secretion was enhanced by 10 ng/ml TGF-β1 in both CRSsNP (n = 7) and control tissue fragments (n = 7) (p<0.05) (Fig 5A). However, TGF-β1 secretion was not enhanced by activin A in the CRSsNP or control tissue fragments (Fig 5B).

## Discussion

**Fig 6:**
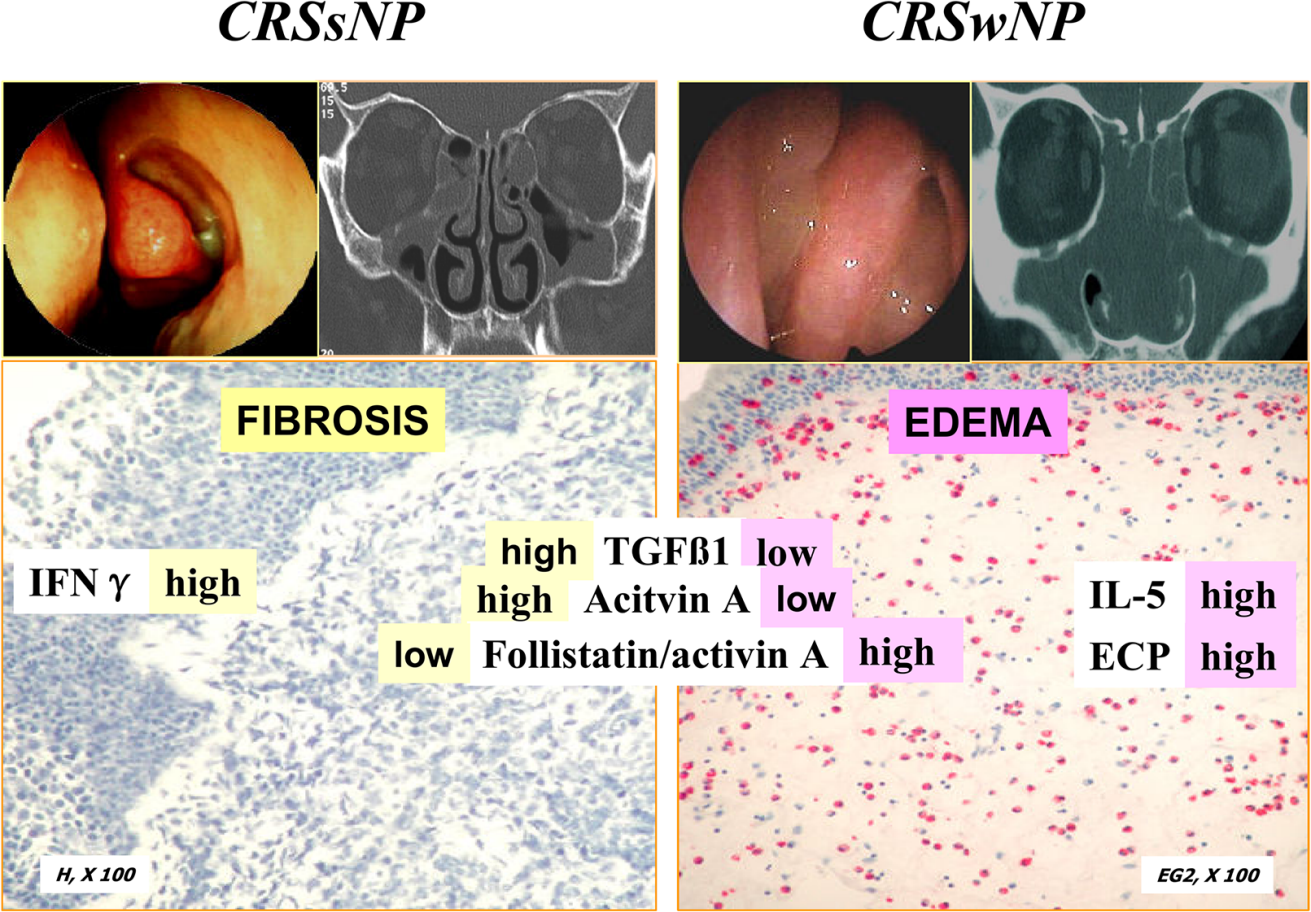
CRSsNP and CRSwNP are two distinct disease entities with respect to remodeling patterns. The hypothesis was supported by the significantly higher protein concentrations of activin A, follistatin, TGF-β1, and IFNγ and the lower concentrations of IL-5 and ECP, lower ratios of follistatin/activinA in CRSsNP compared with CRSwNP tissue homogenates, in line with predominant fibrosis in CRSsNP and edema in CRSwNP.

The results presented here suggest that, along with TGF-β1, activin A and follistatin are cooperative regulators of the remodeling processes during CRS, supporting the hypothesis that CRSsNP and CRSwNP are two distinct disease entities. This idea is consistent with the significantly higher protein concentrations of activin A, follistatin, TGF-β1, and IFNγ and the lower concentrations of IL-5 and ECP in CRSsNP compared with CRSwNP tissue homogenates (Fig 6). Based on the different release patterns of activin A, follistatin and TGF-β1, an imbalance between pro-fibrotic (activin A, TGF-β1) and anti-fibrotic (follistatin) mediators was revealed, which favors fibrosis in CRSsNP and edema in CRSwNP. Furthermore, the fact that TGF-β1 enhanced activin A secretion in CRSsNP tissue fragments indicates that TGF-β1 may potentiate activin A secretion in CRSsNP. Overexpression of activin A and TGF-β1 has been associated with increased lung fibrosis and airway remodeling in the lung [12, 15]. Follistatin, which can bind to and block the actions of activin A, has a beneficial role in attenuating fibrosis [18]. The increased expression of activin A during clinical and experimental asthma suggests that this cytokine is involved in the pathogenesis of asthma [19]. Similarly, these data reveal differential regulation of activin A and follistatin in CRSsNP and CRSwNP, which could be relevant to the pathogenesis of CRS.

The TGF-β superfamily is critically important for tissue development, repair, remodeling and pathogenesis. Continuous crosstalk between TGF-β1 and the members of other signaling pathways, such as activin A, together with factors produced in the tissue micromilieu, such as pro- or anti-inflammatory cytokines, plays an important role in inflammation and remodeling [11, 20]. The previous finding in white patients that CRSsNP is primarily a Th1 response, whereas CRSwNP is associated with Th2 polarization [21], is consistent with the higher protein concentrations of TGF-β1 and IFNγ and the lower concentrations of IL-5 and ECP in CRSsNP compared with CRSwNP tissue homogenates in this study. However, further experiments will be required to determine the molecular mechanism of cooperation between TGF-β1 and these cytokines during airway inflammation and remodeling.

Activin A shares the same cytoplasmic Smad signaling pathway with TGF-β, but it also binds to its own specific transmembrane receptors as well as to follistatin, a secreted protein that inhibits activin by sequestration. Activin A was initially characterized as a reproductive hormone but is now known to play many other roles. Similar to TGF-β1, activin A is a multifunctional cytokine involved in the regulation of tissue remodeling and inflammation, as well as tissue development and repair in various diseases, such as liver fibrosis, pancreatic fibrosis, acute lung injury, asthma, and wound healing [13,14,22]. In a murine model of allergic asthma, follistatin was preformed in the normal lung and then released together with activin A, suggesting that it serves as an endogenous regulator [23]. In a recent study, intranasal follistatin inhibited airway remodeling and decreased airway activin A and TGF-β1 levels in a dose-dependent manner in an ovalbumin-sensitized mouse model [18]. These results demonstrate that follistatin attenuates asthmatic airway remodeling. In our previous studies, enhanced TGF-β1 signaling in CRSsNP and decreased TGF-β1 signaling in CRSwNP were observed, indicating that TGF-β1 is involved in the CRS remodeling process [4, 10]. However, activin A expression and its link to TGF-β-dependent remodeling during CRS are not well understood. In this study, the localization of activin A and follistatin in nasal tissues was identified by immunofluorescence staining, with the epithelial layer been prominent for both molecules. Because the epithelial barrier of CRSwNP has been demonstrated to be leaky [24], the release of these proteins from the tissue through the epithelial barrier may be greater and quicker in CRSwNP vs. CRSsNP. Different tissue remodeling patterns in CRSwNP vs. CRSsNP and the condition of the ECM, impacting on the release of activated TGF-ß1 [25], may further contribute to the dissociation of levels of activin A and follistatin between tissue homogenate protein measurements and their spontaneous release into supernatants at 24 hours.

However, from the differences of the ratios of follistatin/activinA and follistatin/TGF-β1 in tissues and supernatants in CRSsNP and CRSwNP, we can speculate that activin A is more effectively antagonized in CRSwNP than in CRSsNP and that there is an antagonistic effect between follistatin and TGF-β1. Lower activin A protein levels in the tissue homogenates from CRSwNP patients is consistent with the findings from a previous study, in which local decreases in the expression of activin A were shown at sites exhibiting an abundance of B-cell agglomerates in human nasal polyps [26].

The current study demonstrates, for the first time, an interaction between autocrine activin A and TGF-β1 in CRSsNP tissue fragments. The enhancement of activin A secretion by TGF-β1, leading to simultaneously increased expression for both molecules, suggests that activin A and TGF-β1 synergistically promote CRSsNP fibrosis.

Limitations to this study include the lack of PCR data and signaling molecule study, however, we chose for the protein concentrations wherever possible, as RNA does not necessarily get translated into protein, and protein normally is considered the parameter nearer to the actual activity. Furthermore, we relied on historic measurements of fibrosis in the CRS group rather than repeating those measurements in the current study [4, 10, 11]. However, we believe that the new data we present are sufficient to appreciate the findings.

In summary, this study demonstrates a major imbalance in activin A and follistatin expression, similar to that of TGF-β1, in white patients with CRSwNP and CRSsNP. In addition, higher levels of activin A were spontaneously released and its activity was enhanced by TGF-β1 in CRSsNP samples. Considering the observations made by other groups concerning the differential expression of key Th-cell cytokines (e.g., IFNγ and IL-5), these results support the hypothesis that CRSsNP and CRSwNP are distinct disease entities. We also demonstrate that activin A is linked to TGF-β1, and therefore also to remodeling, which is a key feature of CRSsNP. In the network involving activin A and TGF-β1 regulation, activin A appears to be a cooperative regulator and may act as a modulator of the balance between tissue integrity and remodeling processes. A better understanding of the roles of activin A and TGF-β1 in airway disease, as well as the influence of TGF superfamily members as a whole, may provide more specific targets for treatment and prevention. However, specific details concerning the mechanisms of interaction between activin A, TGF-β1 and other cytokines during CRS will require further investigation. An ex-vivo study of the inhibitory effects of follistatin on activin A and TGF-β1 in CRSsNP tissue and primary human sinonasal epithelial cells, and in vivo study of the inhibitory effects on CRSsNP fibrosis are also warranted.

## References

[1] Tomassen P, Newson RB, Hoffmans R, Lotvall J, Cardell LO, Gunnbjörnsdóttir M, et al (2011) Reliability of EP3OS symptom criteria and nasal endoscopy in the assessment of chronic rhinosinusitis—a GA(2) LEN study. Allergy 66:556–561. 10.1111/j.1398-9995.2010.02503.x 21083566

[2] Fokkens WJ, Lund VJ, Mullol J, Bachert C, Alobid I, Baroody F, et al (2012) EPOS 2012: European position paper on rhinosinusitis and nasal polyps 2012. A summary for otorhinolaryngologists. Rhinology 50:1–12. 10.4193/Rhino50E2 22469599

[3] Van Crombruggen K, Zhang N, Gevaert P, Tomassen P, Bachert C. (2011) Pathogenesis of chronic rhinosinusitis: inflammation. J Allergy Clin Immunol 128:728–732. 10.1016/j.jaci.2011.07.049 21868076

[4] Van Bruaene N, Bachert C. (2011) Tissue remodeling in chronic rhinosinusitis. Curr Opin Allergy Clin Immunol 11:8–11. 10.1097/ACI.0b013e32834233ef 21150430

[5] Zhang N, Van Zele T, Perez-Novo C, Van Bruaene N, Holtappels G, DeRuyck N, et al (2008) Different types of T-effector cells orchestrate mucosal inflammation in chronic sinus disease. J Allergy Clin Immunol 122:961–968. 10.1016/j.jaci.2008.07.008 18804271

[6] Bachert C, Zhang N, van Zele T, Gevaert P. (2012) Chronic rhinosinusitis: from one disease to different phenotypes. Pediatr Allergy Immunol 23:2–4. 10.1111/j.1399-3038.2012.01318.x 22762847

[7] Yang YC, Zhang N, Van Crombruggen K, Hu GH, Hong SL, Bachert C. (2012) Transforming growth factor-beta1 in inflammatory airway disease: a key for understanding inflammation and remodeling. Allergy 67:1193–1202. 10.1111/j.1398-9995.2012.02880.x 22913656

[8] Shi M, Zhu J, Wang R, Chen X, Mi L, Walz T, et al (2011) Latent TGF-β structure and activation. Nature 474:343–349. 10.1038/nature10152 21677751 PMC4717672

[9] Yang Y, Zhang N, Lan F, Van Crombruggen K, Fang L, Hu G, et al (2014) Transforming growth factor-beta 1 pathways in inflammatory airway diseases. Allergy 69:699–707. 10.1111/all.12403 24750111

[10] Van Bruaene N, Derycke L, Perez-Novo CA, Gevaert P, Holtappels G, De Ruyck N, et al (2009) TGF-beta signaling and collagen deposition in chronic rhinosinusitis. J Allergy Clin Immunol 124:253–259,e1-2. 10.1016/j.jaci.2009.04.013 19500825

[11] Van Bruaene N, C PN, Van Crombruggen K, De Ruyck N, Holtappels G, Van Cauwenberge P, et al (2012) Inflammation and remodelling patterns in early stage chronic rhinosinusitis. Clin Exp Allergy 42:883–890. 10.1111/j.1365-2222.2011.03898.x 22093003

[12] Hedger MP, de Kretser DM. (2013) The activins and their binding protein, follistatin-Diagnostic and therapeutic targets in inflammatory disease and fibrosis. Cytokine Growth Factor Rev 24:285–295. 10.1016/j.cytogfr.2013.03.003 23541927

[13] Forrester HB, Ivashkevich A, McKay MJ, Leong T, de Kretser DM, Sprung CN. (2013) Follistatin is induced by ionizing radiation and potentially predictive of radiosensitivity in radiation-induced fibrosis patient derived fibroblasts. PLoS One 8:e77119 10.1371/journal.pone.0077119 24204752 PMC3799767

[14] De Kretser DM, O'Hehir RE, Hardy CL, Hedger MP. (2012) The roles of activin A and its binding protein, follistatin, in inflammation and tissue repair. Mol Cell Endocrinol 359:101–106. 10.1016/j.mce.2011.10.009 22037168

[15] Kariyawasam HH, Pegorier S, Barkans J, Xanthou G, Aizen M, Ying S, et al (2009) Activin and transforming growth factor-beta signaling pathways are activated after allergen challenge in mild asthma. J Allergy Clin Immunol 124:454–462. 10.1016/j.jaci.2009.06.022 19733294 PMC4579560

[16] Patou J, Holtappels G, Affleck K, van Cauwenberge P, Bachert C. (2011) Syk-kinase inhibition prevents mast cell activation in nasal polyps. Rhinology 49:100–106. 10.4193/Rhino09.147 21468383

[17] Zhang N, Van Crombruggen K, Holtappels G, Lan F, Katotomichelakis M, Zhang L, et al (2014) Suppression of cytokine release by fluticasone furoate vs. mometasone furoate in human nasal tissue ex-vivo. PLoS One 9:e93754 10.1371/journal.pone.0093754 24710117 PMC3977874

[18] Hardy CL, Nguyen HA, Mohamud R, Yao J, Oh DY, Plebanski M, et al (2013) The activin A antagonist follistatin inhibits asthmatic airway remodelling. Thorax 68:9–18. 10.1136/thoraxjnl-2011-201128 23051972

[19] Kariyawasam HH, Semitekolou M, Robinson DS, Xanthou G. (2011) Activin-A: a novel critical regulator of allergic asthma. Clin Exp Allergy 41:1505–1514. 10.1111/j.1365-2222.2011.03784.x 21631612

[20] Mayer AK, Bartz H, Fey F, Schmidt LM, Dalpke AH. (2008) Airway epithelial cells modify immune responses by inducing an anti-inflammatory microenvironment. Eur J Immunol 38:1689–1699. 10.1002/eji.200737936 18421791

[21] Van Crombruggen K, Van Bruaene N, Holtappels G, Bachert C. (2010) Chronic sinusitis and rhinitis: clinical terminology "Chronic Rhinosinusitis" further supported. Rhinology 48:54–58. 10.4193/Rhin09.078 20502736

[22] Näf S, Escote X, Ballesteros M, Yañez RE, Simón-Muela I, Gil P, et al (2014) Serum activin A and follistatin levels in gestational diabetes and the association of the Activin A-Follistatin system with anthropometric parameters in offspring. PLoS One 9:e92175 10.1371/journal.pone.0092175 24763182 PMC3998926

[23] Hardy CL, O'Connor AE, Yao J, Sebire K, de Kretser DM, Rolland JM, et al (2006) Follistatin is a candidate endogenous negative regulator of activin A in experimental allergic asthma. Clin Exp Allergy 36:941–950. 16839410 10.1111/j.1365-2222.2006.02523.x

[24] Soyka MB, Wawrzyniak P, Eiwegger T, Holzmann D, Treis A, Wanke K, et al (2012) Defective epithelial barrier in chronic rhinosinusitis: the regulation of tight junctions by IFN-γ and IL-4. J Allergy Clin Immunol 130:1087–1096. 10.1016/j.jaci.2012.05.052 22840853

[25] Klingberg F, Chow ML, Koehler A, Boo S, Buscemi L, Quinn TM, et al (2014) Prestress in the extracellular matrix sensitizes latent TGF-β1 for activation. J Cell Biol 207:283–297. 10.1083/jcb.201402006 25332161 PMC4210443

[26] Shoham T, Yaniv E, Koren R, Gal R, Parameswaran R, Kravitz A, et al (2001) Reduced expression of activin A in focal lymphoid agglomerates within nasal polyps. J Histochem Cytochem 49:1245–1252. 11561008 10.1177/002215540104901006

